# PD-L1 expression is associated with epithelial-mesenchymal transition in head and neck squamous cell carcinoma

**DOI:** 10.18632/oncotarget.7431

**Published:** 2016-02-17

**Authors:** Chan-Young Ock, Sehui Kim, Bhumsuk Keam, Miso Kim, Tae Min Kim, Jin-Ho Kim, Yoon Kyung Jeon, Ju-Seog Lee, Seong Keun Kwon, J. Hun Hah, Tack-Kyun Kwon, Dong-Wan Kim, Hong-Gyun Wu, Myung-Whun Sung, Dae Seog Heo

**Affiliations:** ^1^ Department of Internal Medicine, Seoul National University Hospital, Seoul, Korea; ^2^ Department of Pathology, Seoul National University Hospital Seoul, Korea; ^3^ Cancer Research Institute, Seoul National University College of Medicine, Seoul, Korea; ^4^ Department of Radiation Oncology, Seoul National University Hospital, Seoul, South Korea; ^5^ Department of Systems Biology, The University of Texas MD Anderson Cancer Center, Houston, Texas, USA; ^6^ Department of Otorhinolaryngology, Seoul National University Hospital, Seoul, South Korea

**Keywords:** PD-L1, epithelial-mesenchymal transition, head and neck, squamous, p16

## Abstract

Virus-associated malignancies and sarcomatoid cancers correlate with high PD-L1 expression, however, underlying mechanisms remain controversial. We evaluated the correlation between PD-L1 expression and epithelial-mesenchymal transition (EMT) in head and neck squamous cell carcinomas (HNSCC). Tumor tissues from 50 patients with HNSCC were evaluated for PD-L1 by immunohistochemistry, which showed 32 (64.0%) were PD-L1 positive (PD-L1+). Interestingly, PD-L1 expression was significantly associated with EMT (*P* = 0.010), as assessed by low E-cadherin and high vimentin expression. The overall survival of PD-L1+ patients with EMT features was significantly worse than those without EMT features (*P* = 0.007). In an independent validation cohort (N = 91), as well as in HNSCC cases of The Cancer Genome Atlas (TCGA) and the Cancer Cell Line Encyclopedia, high PD-L1 expression was also associated with the high probability of an EMT signature, referred from the GEO dataset, GSE4824. Survival analysis confirmed PD-L1+/EMT+ patients had a poorer prognosis than PD-L1+/EMT- patients in the TCGA cohort. PD-L1 positivity can thus be divided into two categories according to the absence or presence of EMT. PD-L1 expression is also independently associated with EMT features in HNSCC.

## INTRODUCTION

Discovery of programmed death-ligand 1 (PD-L1) expression in tumors has encouraged research toward more efficient immunologic methods of conquering cancer [1]. Cancer cells have developed various strategies for evading host anti-cancer immunologic attacks, including the up-regulation of PD-L1, which induces T cell anergy and apoptosis by interacting with programmed death-1 (PD-1) receptors [2, 3]. The development of immune checkpoint inhibitors that block PD-1/PD-L1 interaction has been clinically successful, with a long response time noted [4, 5]. However, more than half of patients evaluated were insensitive to these agents, highlighting the importance of distinguishing between those who may be sensitive or resistant to such inhibitors. Various biomarker candidates may predict responses to anti-PD-1/PD-L1 blocking agents, with PD-L1 expression in tumors a leading prospect [6–8].

Viruses and epithelial-mesenchymal transition (EMT) are associated with high PD-L1 expression [9]. PD-L1 up-regulation is observed in Epstein-Barr virus (EBV)-associated malignancies such as lymphoma, nasopharyngeal and stomach cancer. PD-L1 up-regulation also occurs in cancers associated with human papillomavirus (HPV) such as uterine, cervical, head and neck cancers [10–15]. PD-L1 up-regulation may occur in response to the constitutional up-regulation of *CD274* gene amplification at 9p24.1, which encodes PD-L1 [10]. Another possible mechanism is PD-L1 induction by interferon-gamma secreted from tumor-infiltrating immune cells via the JAK/STAT pathway [12–16]. PD-L1 expression is also associated with the mesenchymal signature of tumors. For example, more than half of sarcoma patients showed high PD-L1 expression levels, irrespective of tumor type [17]. Moreover, PD-L1 was highly expressed in 69.2% of sarcomatoid lung carcinomas [18]. EMT changes, manifested by E-cadherin (encoded by *CDH1*) down-regulation and vimentin (encoded by *VIM*) up-regulation, also correlated with PD-L1 induction [19]. However, a comprehensive analysis of the association of PD-L1 with viruses and EMT has not yet been reported.

Head and neck squamous cell carcinoma (HNSCC) is a suitable model to investigate the clinicopathologic features associated with PD-L1 up-regulation. Recent advances in genomics have shown that mutational HPV-positive and -negative tumor profiles, which have retained and lost p16 expression, respectively, clearly differ [20–22]. Although HPV/p16-positive tumors showed high PD-L1 expression compared to HPV/p16-negative tumors [14, 23, 24], the statistical significance between PD-L1 and HPV was not strong. In addition, a significant proportion of HPV/p16-negative tumors also showed high PD-L1 expression. Interestingly, 19.2% to 37.4% of HNSCC cases exhibited inflamed/mesenchymal features; however, this cluster was not definitively correlated with HPV/p16 positivity [25]. Therefore, PD-L1 expression associated with HPV/p16 and EMT is of interest.

In the current study, we analyzed 1) PD-L1 expression according to the EMT and p16 statuses of patients with HNSCC, and 2) their clinical implications. A gene expression signature associated with EMT was obtained from the publicly available database, GEO4824 [26]. This signature was then applied to The Cancer Genome Atlas (TCGA) database [20] and the Cancer Cell Line Encyclopedia (CCLE) [27] to validate the significance of the correlation between PD-L1 and EMT in independent cohorts.

## RESULTS

### PD-L1 expression correlated with EMT

**Figure 1:**
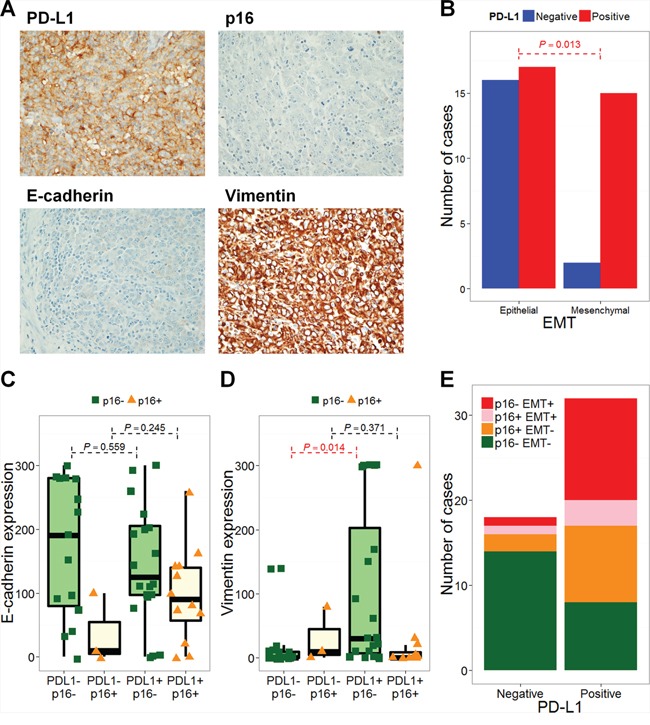
PD-L1 expression is associated with epithelial-mesenchymal transition Representative images of immunohistochemical staining with anti-PD-L1 (× 200; **A.** × 400, **B.**), anti-E-cadherin (× 200, **C.**), and anti-vimentin (× 200, **D.**) are shown. PD-L1 expression was positively correlated with vimentin and negatively correlated with E-cadherin. The number of PD-L1-negative (blue bars) and -positive (red bars) cases according to epithelial-mesenchymal transition (EMT) are shown. The *P* value from Fisher's exact test is annotated (red). H-scores for E-cadherin (C) and vimentin (D) are plotted according to PD-L1 and p16 statuses. The number of p16- EMT- (green), p16+ EMT- (orange), p16+ EMT+ (pink), and p16- EMT+ (red) cases according to PD-L1 status are shown **E.**

Representative images of PD-L1-negative and -positive, p16-positive and high E-cadherin expressing tumor tissues are shown in Supplementary Figure 1. Of the 50 patients included in this study (training cohort), 32 and 18 patients, respectively, exhibited PD-L1-positive and –negative tumors. Fifteen patients were p16-positive and 17 were EMT-positive. Interestingly, 15 of the 32 PD-L1-positive tumors (46.9%) were EMT-positive, as assessed by low E-cadherin and high vimentin expression (Figure 1A). PD-L1 positivity was significantly higher in patients with EMT-positive tumors (*P* = 0.013, Figure 1B). Clinical features, including age, sex, smoking history, and stage, did not differ according to patients' PD-L1 and EMT statuses (Table 1, left column). The proportion of patients showing oropharyngeal tumors was higher in PD-L1-positive/EMT-negative patients (PD-L1+/EMT-; Table 1, left column). The E-cadherin H-score was lower for PD-L1+/p16- compared with PD-L1-/p16- patients (*P* = 0.559). The vimentin H-score was significantly higher in PD-L1+/p16- compared with PD-L1-/p16- patients (*P* = 0.014). However, this trend was not observed in p16+ patients (*P* = 0.245 and 0.371, respectively; Figure 1C-1D). Taken together, of the 32 PD-L1+ patients, 12 were p16-/EMT+ (37.5%), 9 were p16+/EMT- (28.1%), 8 were p16-/EMT- (25%), and 3 were p16+/EMT+ (9.4%; Figure 1E). Although PD-L1 positivity was not significantly different according to p16 status (*P* = 0.199; Supplementary Figure 2A), PD-L1 positivity was significantly higher in p16-/EMT+ and p16+/EMT- compared with p16-/EMT- patients (*P* = 0.002 and 0.026, respectively; Supplementary Figure 2B). Univariate and multivariate logistic regression analyses showed that an oropharyngeal tumor origin and EMT status associated significantly with PD-L1 positivity (*P* = 0.014 and 0.010, respectively; Table 2, left column).

**Table 1:** Patient characteristics

		Training cohort					Validation cohort				
		AllN=50	PD-L1(−) N=18	PD-L1(+) EMT(−) N=17	PD-L1(+) EMT(+) N=15	*P* value	AllN=91	PD-L1(−) N=32	PD-L1− LI(+) EMT(−) N=32	PD-L1(+) EMT(+) N=27	*P* value
Age	Median years(range)	60(16-78)	61(44-78)	57(16-75)	61(26-76)	0.074	59(20-89)	61(20-89)	59(29-80)	59(31-79)	0.853
Sex	Men, N (%)	40 (80.0)	14 (77.8)	15 (88.2)	11 (73.3)		61 (67.0)	25 (78.1)	19 (59.4)	17 (63.0)	
	Women, N (%)	10 (20.0)	4 (22.2)	2 (11.8)	4 (26.7)	0.614	30 (33.0)	7 (21.9)	13 (40.6)	10 (37.0)	0.232
Smoking	Non-smoker, N (%)	30 (60.0)	10 (55.6)	10 (58.8)	10 (66.7)		65 (71.4)	22 (68.8)	26 (81.3)	17 (63.0)	
	Ex/Current-smoker, N (%)	20 (40.0)	8 (44.4)	7 (41.2)	5 (33.3)	0.877	26 (28.6)	10 (31.3)	6 (18.8)	10 (37.0)	0.281
ECOG	0, N (%)	12 (24.0)	2 (11.1)	6 (35.3)	4 (26.7)		53 (58.2)	18 (56.3)	17 (53.1)	18 (66.7)	
	1, N (%)	38 (76.0)	16 (88.9)	11 (64.7)	11 (73.3)	0.243	38 (41.8)	14 (43.8)	15 (46.9)	9 (33.3)	0.599
Location	Oropharynx, N (%)	16 (32.0)	2 (11.1)	10 (58.8)	4 (26.7)		39 (42.9)	9 (28.1)	19 (59.4)	11 (40.7)	
	Non-oropharynx*, N (%)	34 (68.0)	16 (88.9)	7 (41.2)	11 (73.3)	**0.010**	52 (57.1)	23 (71.9)	13 (40.6)	16 (59.3)	**0.040**
p16	Negative, N (%)	35 (70.0)	15 (83.3)	8 (47.1)	12 (80.0)		58 (63.7)	23 (71.9)	17 (53.1)	18 (66.7)	
	Positive, N (%)	15 (30.0)	3 (16.7)	9 (52.9)	3 (20.0)	0.051	33 (36.3)	9 (28.1)	15 (46.9)	9 (33.3)	0.312
Pathology	P/D, N (%)	24 (48.0)	8 (44.4)	11 (64.7)	5 (33.3)		25 (27.5)	6 (18.8)	9 (28.1)	10 (37.0)	
	M/D, N (%)	12 (24.0)	6 (33.3)	3 (17.7)	3 (20.0)		37 (40.7)	14 (43.8)	11 (34.4)	12 (44.4)	
	W/D, N (%)	12 (24.0)	4 (22.2)	2 (11.8)	6 (40.0)		29 (31.9)	12 (37.5)	12 (37.5)	5 (18.5)	
	Non-keratinizing type, N (%)	2 (4.0)	0 (0.0)	1 (5.9)	1 (6.7)	0.331	0 (0)	0 (0)	0 (0)	0 (0)	0.342
Stage	I, N (%)	6 (12.0)	1 (5.6)	1 (5.9)	4 (26.7)		11 (12.2)	6 (19.4)	3 (9.4)	2 (7.4)	
	II, N (%)	2 (4.0)	0 (0.0)	1 (5.9)	1 (6.7)		16 (17.8)	7 (22.6)	4 (12.5)	5 (18.5)	
	III, N (%)	15 (30.0)	9 (50.0)	5 (29.4)	2 (13.3)		16 (17.8)	3 (9.7)	6 (18.8)	7 (25.9)	
	IVA, N (%)	27 (54.0)	8 (44.4)	10 (58.8)	8 (53.3)	0.253	47 (52.2)	15 (48.4)	19 (59.4)	13 (48.2)	0.504
Definitive Treatment	Concurrent chemoradiotherapy, N (%)	16 (32.0)	7 (38.9)	4 (23.5)	5 (33.3)		11 (12.1)	2 (6.3)	7 (21.9)	2 (7.4)	
	Surgery, N (%)	34 (68.0)	11 (61.1)	13 (76.5)	10 (66.7)	0.693	80 (87.9)	30 (93.8)	25 (78.1)	25 (92.6)	0.167
Overall survival	Median months (95% CI)	NR(43.7-NR)	50.1(25.0-NR)	NR(NR-NR)	35.7(30-NR)	0.007**	117(102.4-NR)	117(102.4-NR)	NR(NR-NR)	26.3(11.2-NR)	0.003**
	3-year survival rate	73.7%	74.3%	100%	42.8%		71.0%	83.6%	80.3%	45.6%	
	5-year survival rate	53.2%	41.3%	100%	21.4%		68.1%	77.6%	80.3%	45.6%	
Median follow-up	Median months (range)	72.4(23.0-119.6)	84.5(27.4-119.6)	50.3(33.0-112.7)	48.2(23-112.7)	0.623	32.2(6.3-234.6)	43.1(7.3-234.6)	24.5(9.1-160)	32.2(6.3-150.3)	0.178

Bold values indicate statistically significant correlations with *P* values less than 0.05.

* Non-oropharynx included hypopharynx, larynx, nasal cavity, paranasal sinus, oral cavity, and nasopharynx tumors, which were not significant according to PD-L1 positivity.

** Log rank *P* value comparing 3 groups

Abbreviation: EMT, epithelial-mesenchymal transition; ECOG, Eastern Cooperative Oncology Group performance status; P/D, poorly-differentiated squamous cell carcinoma; M/D; moderate-differentiated squamous cell carcinoma; W/D, well-differentiated squamous cell carcinoma; CI, confidence interval; NR, not reached.

**Table 2:** Univariate and multivariate logistic analysis of factors affecting PD-L1 expression

		Training cohort				Validation cohort			
		Univariate		Multivariate		Univariate		Multivariate	
		HR (95% CI)	*P* value	HR (95% CI)	*P* value	HR (95% CI)	*P* value	HR (95% CI)	*P* value
Age	Continuous	0.97 (0.92-1.02)	0.275			0.99 (0.96-1.03)	0.646		
Sex	Female (vs. Male)	0.81 (0.19-3.35)	0.769			2.28 (0.85-6.13)	0.102		
Smoking	Yes (vs. No)	0.75 (0.23-2.42)	0.631			0.82 (0.32-2.10)	0.677		
ECOG	1 (vs. 0)	0.28 (0.05-1.43)	0.125			0.88 (0.37-2.11)	0.777		
Stage	Continuous	0.81 (0.44-1.49)	0.495			1.34 (0.90-1.99)	0.151		
Location	Oropharynx (vs. Non-oropharynx)	6.22 (1.22-31.7)	**0.028**	8.60 (1.54-48.1)	**0.014**	2.64 (1.05-6.66)	**0.039**	3.63 (1.33-9.90)	**0.012**
p16	Positive (vs. Negative)	3.00 (0.72-12.5)	0.132			1.75 (0.69-4.44)	0.237		
EMT	Positive (vs. Negative)	7.06 (1.39-35.9)	**0.018**	9.54 (1.72-52.9)	**0.010**	4.56 (1.54-13.5)	**0.006**	5.96 (1.90-18.7)	**0.002**

Bold values indicate statistically significant correlations with *P* values less than 0.05.

Abbreviation: ECOG, Eastern Cooperative Oncology Group performance status; HR, hazard ratio; CI, confidence interval; EMT, epithelial-mesenchymal transition.

Training cohort findings were confirmed in the independent validation cohort (N = 91), where PD-L1 positivity was significantly higher in patients with EMT-positive tumors (PD-L1+ in EMT- versus EMT+: 54.2% versus 84.4%; *P* = 0.003). The number of patients allocated to PD-L1-, PD-L1+/EMT-, and PD-L1+ /EMT+ groups were 32, 32, and 27, respectively (Table 1, right column). Univariate and multivariate logistic regression analyses of the validation cohort also showed that oropharyngeal tumor origin and EMT status were significantly associated with PD-L1 positivity (*P* = 0.012 and 0.002, respectively; Table 2, right column).

### Survival analysis according to PD-L1 and EMT statuses

**Figure 2:**
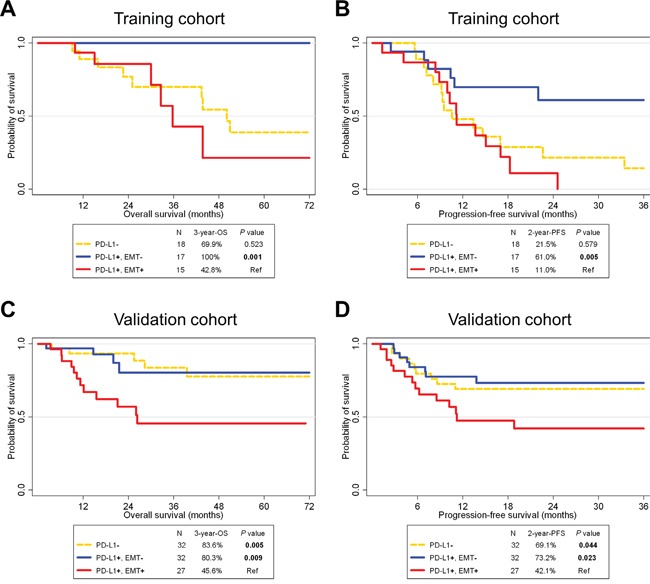
Survival analysis according to PD-L1 and epithelial-mesenchymal transition statuses in HNSCC patients of training (A, B) and validation cohorts (C, D) A Kaplan-Meier plot of overall survival (OS; A, C) and progression-free survival (PFS; B, D) according to PD-L1 and epithelial-mesenchymal transition (EMT) statuses. Abbreviations: HNSCC, head and neck squamous cell carcinoma; Ref, reference.

Survival analysis according to PD-L1 expression in HNSCC has not been clearly defined. In both training and validation cohorts, PD-L1 expression was not significantly associated with overall survival (OS; training cohort, *P* = 0.137, and validation cohort, *P* = 0.202) or progression-free survival (PFS; training cohort, *P* = 0.213, and validation cohort, *P* = 0.494; Supplementary Figure 3). In regard to the training cohort, interestingly, PD-L1+/EMT+ patients showed significantly poorer OS and PFS rates compared to PD-L1+/EMT- patients (*P* < 0.001 and 0.005, respectively; Figure 2A-2B). The 3-year OS rate was 42.8% for PD-L1+/EMT+ patients, which differed markedly from 100% for PD-L1+/EMT- patients. Moreover, PD-L1+/EMT+ patients showed significantly poorer OS and PFS rates compared with PD-L1+/p16+/EMT- patients (*P* = 0.007 and 0.006, respectively; Supplementary Figure 4A-4B). Univariate Cox regression analysis indicated that tumor location and EMT status significantly correlated with the OS rate. However, only the EMT status was significant in multivariate analysis (adjusted hazard ratio 2.82, 95% confidence interval 1.01–7.94; *P* = 0.049; Table 3, left column). These trends on the prognostic impacts of PD-L1+/EMT+ were also significant in the validation cohort: PD-L1+/EMT+ was associated with worse OS and PFS rates compared with PD-L1+/EMT- (*P* = 0.009 and 0.023, respectively; Figure 2C-2D) or PD-L1+/p16+/EMT- (*P* = 0.047 and 0.013, respectively; Supplementary Figure 4C-4D). Multivariate Cox regression analysis for the validation cohort revealed that performance and EMT status both significantly correlated with the OS rate (adjusted *P* = 0.007 and 0.002, respectively; Table 3, right column; see also Supplementary Table 2 for PFS analysis).

**Table 3:** Univariate and multivariate Cox regression analysis of factors affecting overall survival

		Training cohort				Validation cohort			
		Univariate		Multivariate		Univariate		Multivariate	
		HR (95% CI)	*P* value	HR (95% CI)	*P* value	HR (95% CI)	*P* value	HR (95% CI)	*P* value
Age	Continuous	1.01 (0.96-1.06)	0.660			1.01 (0.98-1.05)	0.373		
Sex	Female (vs. Male)	0.92 (0.26-3.27)	0.898			1.89 (0.83-4.29)	0.129		
Smoking	Yes (vs. No)	1.67 (0.60-4.61)	0.322			0.79 (0.29-2.15)	0.648		
ECOG	1 (vs. 0)	0.93 (0.26-3.35)	0.916			2.60 (1.14-5.97)	**0.024**	3.14 (1.36-7.26)	**0.007**
Stage	Continuous	1.66 (0.82-3.37)	0.158			0.84 (0.59-1.19)	0.318		
Location	Oropharynx (vs. Non-oropharynx)	0.22 (0.05-0.97)	**0.046**	0.25 (0.06-1.10)	0.067	0.43 (0.17-1.10)	0.077		
PD-L1	Positive (vs. Negative)	0.46 (0.16-1.31)	0.148			1.78 (0.73-4.34)	0.206		
p16	Positive (vs. Negative)	0.50 (0.16-1.61)	0.246			0.47 (0.18-1.26)	0.135		
EMT	Positive (vs. Negative)	3.19 (1.12-9.07)	**0.029**	2.82 (1.01-7.94)	**0.049**	3.48 (1.44-8.41)	**0.006**	4.15 (1.70-10.1)	**0.002**

Bold values indicate statistically significant correlations with *P* values less than 0.05.

Abbreviation: ECOG, Eastern Cooperative Oncology Group performance status; HR, hazard ratio; CI, confidence interval; EMT, epithelial-mesenchymal transition.

### Correlation of PD-L1 with EMT in TCGA and CCLE

**Figure 3:**
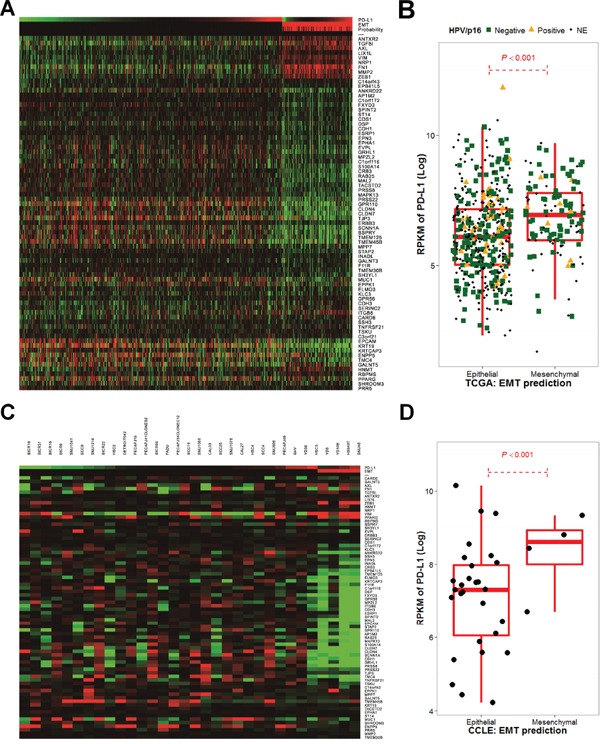
The epithelial-mesenchymal transition gene expression signature correlates with PD-L1 expression in The Cancer Genome Atlas and the Cancer Cell Line Encyclopedia Significant 75-gene expression signatures, referred from GSE4824 and Bayesian probability to predict EMT changes that favor mesenchymal features in The Cancer Genome Atlas (TCGA) **A.** and the Cancer Cell Line Encyclopedia (CCLE) cohorts **C.** as well as PD-L1 expression, are shown as a heatmap. PD-L1 expression was calculated by the log 2 value of its reads per kilobase of transcript per million mapped reads (RPKM) and was compared according to EMT predictions in TCGA **B.** and CCLE cohorts **D.**

A 75 gene EMT expression signature was obtained from the GSE4824 dataset, which predicted mesenchymal features in lung cancer cell lines, including squamous cell carcinoma [26]. EMT predictions based on the 75 gene signature were conducted for HNSCC samples and cell lines from TCGA and CCLE, respectively [20, 27]. EMT predictions resulted in 119 out of 564 HNSCC samples (21.1%) from TCGA, and 4 out of 32 cell lines (12.5%) from CCLE having mesenchymal features (Figure 3A, 3C). Samples with a high probability of mesenchymal features exhibited a high expression of mesenchymal signatures, such as *ZEB1* and *VIM* that encode vimentin, and low expression of epithelial signatures, such as *MUC1* and CDH1 that encode E-cadherin. Interestingly, in both TCGA and CCLE, PD-L1 expression was significantly higher in mesenchymal features compared with epithelial features (*P* < 0.001; Figure 3B, 3D). The HPV/p16 status was disclosed in 277 cases (221 HPV/p16- and 56 HPV/p16+) from TCGA samples [20]. PD-L1 expression did not differ according to HPV/p16 status (*P* = 0.651). However, p16-/EMT+ displayed significantly higher PD-L1 expression compared to p16-/EMT- or p16+/EMT- (*P* < 0.001 and 0.012, respectively; Supplementary Figure 5).

**Figure 4:**
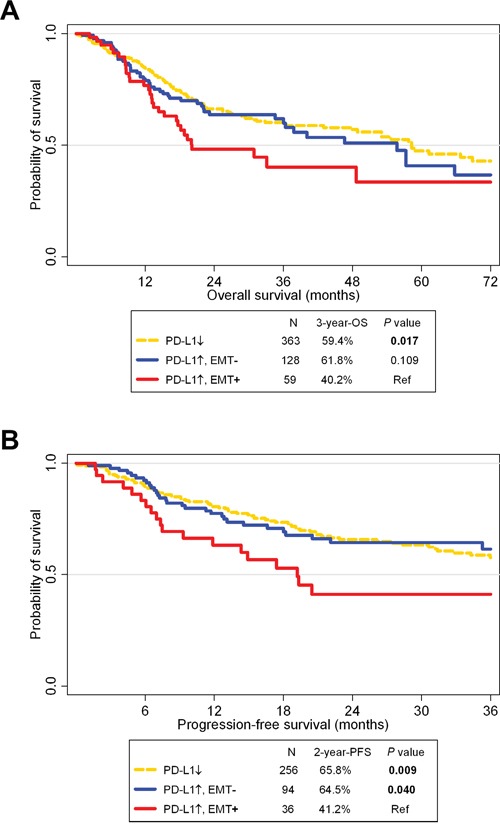
Survival analysis according to PD-L1 and epithelial-mesenchymal statuses in The Cancer Genome Atlas cohort A Kaplan-Meier plot of overall survival (OS; **A.**) and progression-free survival (PFS; **B.**) according to PD-L1 expression and epithelial-mesenchymal transition (EMT) status. Abbreviation: Ref, reference.

OS and PFS rates for PD-L1+/EMT+ patients were significantly worse compared to those for PD-L1- patients from TCGA (*P* = 0.017 and 0.009, respectively; Figure 4). PFS rates were significantly different between PD-L1+/EMT+ and PD-L1+/EMT- patients (*P* = 0.040). However, OS rates were not significantly different, although the 3-year OS rate differed between PD-L1+/EMT+ (40.2%) and PD-L1+/EMT- (61.8%; *P* = 0.109).

## DISCUSSION

We investigated the clinical significance of EMT and p16 on PD-L1 expression in HNSCC. PD-L1 expression is independently associated with mesenchymal features. Correlations between PD-L1 and EMT were also validated in an independent validation cohort as well as in public TCGA and CCLE databases using an EMT gene expression signature. Patients who were PD-L1+/EMT+ showed a significantly poorer prognosis than those who were PD-L1+/EMT-. This finding suggests that PD-L1 positivity can be divided into two categories by EMT markers in HNSCC.

Together with the mutational burden of tumors [28] and an abundance of tumor-infiltrating lymphocytes (TILs) [29], PD-L1 expression in tumor tissues or niches strongly correlates with response rates to anti-PD-1/PD-L1 inhibitors [6, 7]. However, the specific clinicopathologic factors associated with PD-L1 expression in cancer are unclear. This is because multi-factorial influences on PD-L1 expression exist including viruses, EMT changes, and sub-lethal damage induced by cytotoxic chemotherapy via MAPK or JAK/STAT pathways [9, 15, 19, 30, 31]. Although an association between HPV and PD-L1 expression has been reported [14], a major proportion of HNSCC cases exhibit an inflamed/mesenchymal signature that is independent of HPV status [21]. In the current study, PD-L1 expression significantly correlated with EMT rather than HPV/p16 status. In addition to an association between PD-L1 and EMT in a rodent model [19] and a strong PD-L1 association with cancers of mesenchymal origin [17, 18], this is the first report to show a correlation between PD-L1 and EMT in cancers that originate from epithelial tissue. This correlation was significant both in clinical samples from a cohort of Korean patients and in an independent cohort that primarily consisted of Western patients in TCGA [20] and a comprehensive cancer cell line database, CCLE [27].

A statistical significance regarding PD-L1 associating with HPV/p16 status was not observed in clinical or TCGA samples. However, HPV/p16 could also partially contribute to PD-L1 expression given that oropharyngeal tumors, with their high prevalence of HPV infection, also correlated with PD-L1 positivity. HPV-positive HNSCC and other virus-associated cancers, such as HPV-positive uterine cervical cancer and EBV-positive gastric cancer, show increased immunogenic features such as an abundance of TIL or CD8-positive cytotoxic T cell signatures [32, 33]. Because the correlation between PD-L1 and EMT is increased in HPV/p16-negative patients, HPV/p16-positivity and EMT features could contribute to PD-L1 expression in a mutually exclusive manner.

Overall prognosis according to PD-L1 status is controversial due to various factors. PD-L1 expression is considered a poor prognostic factor in cancers such as non-small cell lung cancer, renal cell carcinoma, and melanoma [34–36]. In contrast, PD-L1 status is a good prognostic factor in colorectal cancer [37]. However, in regard to HNSCC, the lack of information in the literature concerning its prognosis according to PD-L1 expression could be due to a bias that PD-L1 is not important in this disease. In the current study, PD-L1 expression did not solely affect significant survival differences. However, the inclusion of factors associated with PD-L1 up-regulation, such as HPV/p16 positivity and EMT features, clearly defined distinct patient groups by survival differences. In PD-L1-positive patients, p16 positivity conferred a much better prognosis than EMT positivity given that viral associations induce immunogenic features in tumors, which confer a good prognosis in HNSCC [38, 39]. Cancers showing EMT are associated with early recurrence and aggressive metastases [40]; therefore, EMT-associated PD-L1-positive cancers have a poor prognosis. This prognostic trend was validated in a TCGA cohort that included diverse ethnicities.

Investigating upstream mechanisms of PD-L1 up-regulation and their clinical impacts is challenging. PD-L1 expression is induced by extrinsic stimuli such as interferon-gamma [1] produced by the inflamed niche surrounding tumors in response to viral associations or neo-antigen processing [9, 41]. However, PD-L1 expression is also induced by the activation of intrinsic oncogenic pathways, such as STAT3, an activating EGFR mutation or ALK translocation [42, 43]; together with EMT, these share similar molecular pathways and EMT-associated genes. Additionally, because the clinical implications of PD-L1 up-regulation differ depending on intrinsic and extrinsic induction mechanisms, their predictive values with anti-PD-1/PD-L1 inhibitors should be investigated, particularly the predictive values of PD-L1+/EMT+ and PD-L1+/EMT- statuses.

The retrospective design and relatively small number of samples with a heterogeneous clinical status used in the current study could have biased results. Since we aimed to find an association between PD-L1 and EMT in a clinical setting, patients with nasopharyngeal cancer (N = 1) and paranasal sinus cancer (N = 2) were included; of these, two patients showed a non-keratinizing type pathology. Therefore, caution is needed in the direct application of the current results to further clinical studies. However, significant statistical results were obtained for the independent validation cohort, as well as for TCGA and the CCLE open databases. The clinical outcomes of anti-PD-1/PD-L1 inhibitor therapy is lacking in this study, although overall prognosis according to PD-L1 expression and its up-stream mechanism should be understood before researching clinical outcomes of the target agent. Although the prognostic impact of PD-L1 expression was not reported, we clearly showed PD-L1 up-regulation according to two distinct up-stream pathways, EMT and HPV/p16, influenced the overall prognosis. The association of EMT with PD-L1 in human cancer is a novel finding in this study.

In conclusion, PD-L1 expression is associated with EMT, an independent up-stream pathway distinct from HPV/p16 association. EMT-associated PD-L1 expression confers a significantly poorer prognosis compared to PD-L1 expression not associated with EMT. Clinical investigations using anti-PD-1/PD-L1 inhibitors in patients with EMT-associated PD-L1 up-regulation are warranted.

## MATERIALS AND METHODS

### Study population

Medical records were retrospectively reviewed for patients diagnosed with locally advanced HNSCC and treated at Seoul National University Hospital between December 2004 and November 2012. Fifty patients with paraffin-embedded tumor samples were included. Firstly, we analysed the association of PD-L1 and EMT in 50 HNSCC patients (training cohort). To confirm preliminary findings, we enrolled a further 91 HNSCC patients (validation cohort), and performed similar analyses.

### Treatment

Treatment decisions were determined by a multidisciplinary team [44, 45]. Patients were treated with definitive concurrent chemoradiotherapy (CCRT) or radical surgery, including primary tumor and regional lymph node dissection. The CCRT regimen consisted of weekly cisplatin. Radiation therapy was provided as a standard fractionated dose of more than 60 Gy for primary tumors and regional lymph nodes, with concurrent cisplatin chemotherapy.

### Immunohistochemistry

Representative, formalin-fixed, paraffin-embedded tissue blocks from each case were submitted for immunohistochemistry (IHC) using the following antibodies: mouse anti-p16 (E6H4) monoclonal antibody (mAb; Roche/MTM/Ventana Medical Systems, Tucson, AZ, USA), mouse anti-E-cadherin (36B5) mAb (Novocastra Laboratories, Newcastle upon Tyne, UK), mouse anti-vimentin (V9) mAb (Dako, Ely, UK) and rabbit anti-PD-L1 (E1L3N) XP^®^ mAb (Cell Signaling Technology, Danvers, MA, USA). IHC was performed using the Ventana Benchmark XT system (Ventana Medical Systems). When tissue sections showed diffuse and strong nuclear and cytoplasmic staining in ≥ 70% of tumor cells, this was considered positive for p16 [46]. For E-cadherin and vimentin, staining intensity was scored in four categories: no staining (0), weak (1+), moderate (2+) and strong (3+) staining. The percentage of tumor cells showing the different staining intensities were evaluated by a trained pathologist. An IHC score (H-score) was then calculated using the following formula: 1 × (percentage of cells showing weak staining) + 2 × (percentage of cells showing moderate staining) + 3 × (percentage of cells showing strong staining). E-cadherin and vimentin were used as an epithelial or mesenchymal phenotype marker, respectively. An EMT phenotype was defined as low E-cadherin expression with an H-score < 200 and high vimentin expression with a H-score > 30. PD-L1 IHC was evaluated based on the intensity and proportion of membranous staining, with or without cytoplasmic staining, in tumor cells and was scored as follows: 0, less than 5% of tumor cells; 1, weak in ≥ 5% of tumor cells; 2, moderate in ≥ 5% of tumor cells; and 3, strong in ≥ 5% of tumor cells. Cases showing membranous staining for PD-L1 in ≥ 5% of tumor cells (i.e., including IHC scores 1, 2 or 3) were considered PD-L1-positive.

### Statistical analyses of microarray, RNA sequencing, and clinicopathologic data

The BRB-ArrayTools software program (http://linus.nci.nih.gov/BRB-ArrayTools.html) was used in the analysis of gene expression data [47]. A heatmap was generated using Cluster and TreeView software programs [48]. To assess EMT characteristics of tumors from an HNSCC cohort of TCGA study [20] and cell lines from CCLE [27], 75 gene expression signatures associated with EMT from a previous study, GSE4824 [26], and a previously developed approach were used [49, 50]. Briefly, the 75 gene expression data in the training set (GSE35640, Supplementary Table 1) were combined to form a classifier according to a Bayesian compound covariate predictor (BCCP) [51]. The robustness of the classifier was assessed using a misclassification rate determined during leave-one-out cross-validation in the training set. The BCCP classifier estimated the likelihood that an individual patient had either an epithelial or mesenchymal signature according to a Bayesian probability cut-off of 0.5, which was optimized by comparing results with a previously reported proportion of EMT signatures in HNSCC [25]. A comparison of continuous values, such as H-scores for E-cadherin and vimentin, or the log 2 value of reads per kilobase of transcript per million mapped reads (RPKM) of PD-L1, was undertaken using a Wilcoxon rank sum test. A fisher's exact test was used to determine associations between clinicopathologic parameters. The significance of clinicopathologic factors on PD-L1-positivity was calculated by logistic regression. OS was measured from the diagnosis date until death, or the last follow-up date if censored. Progression-free survival (PFS) was calculated from the first day of definitive treatment up to the date of disease progression, confirmed by imaging, death, or the last follow-up date if censored. Survival analyses were carried out according to the Kaplan-Meier method with log-rank testing to assess differences between groups. A Cox proportional hazard regression model was used for univariate and multivariate survival analyses. The RPKM cut-off for PD-L1 in survival analyses was determined by a median value of PD-L1 RPKM for mesenchymal signatures. All reported *P* values were two-sided, and considered significant if *P* < 0.05. A false discovery rate were applied to control type I errors. All statistical analyses and data generation were carried out using R version 3.1.3 (http://www.r-project.org) and STATA version 12 (StataCorp LP, College Station, TX, USA).

## SUPPLEMENTARY FIGURES AND TABLES




