# Supplementary material for: PD-L1 expression is associated with epithelial-mesenchymal transition in head and neck squamous cell carcinoma
Source: Oncotarget. 2016 Feb 17;7(13):15901–14. doi: 10.18632/oncotarget.7431 (PMC4941285; doi:10.18632/oncotarget.7431)
Supplement: Supplementary file 1 [file oncotarget-07-15901-s001.pdf]

SUPPLEMENTARY FIGURES AND TABLES

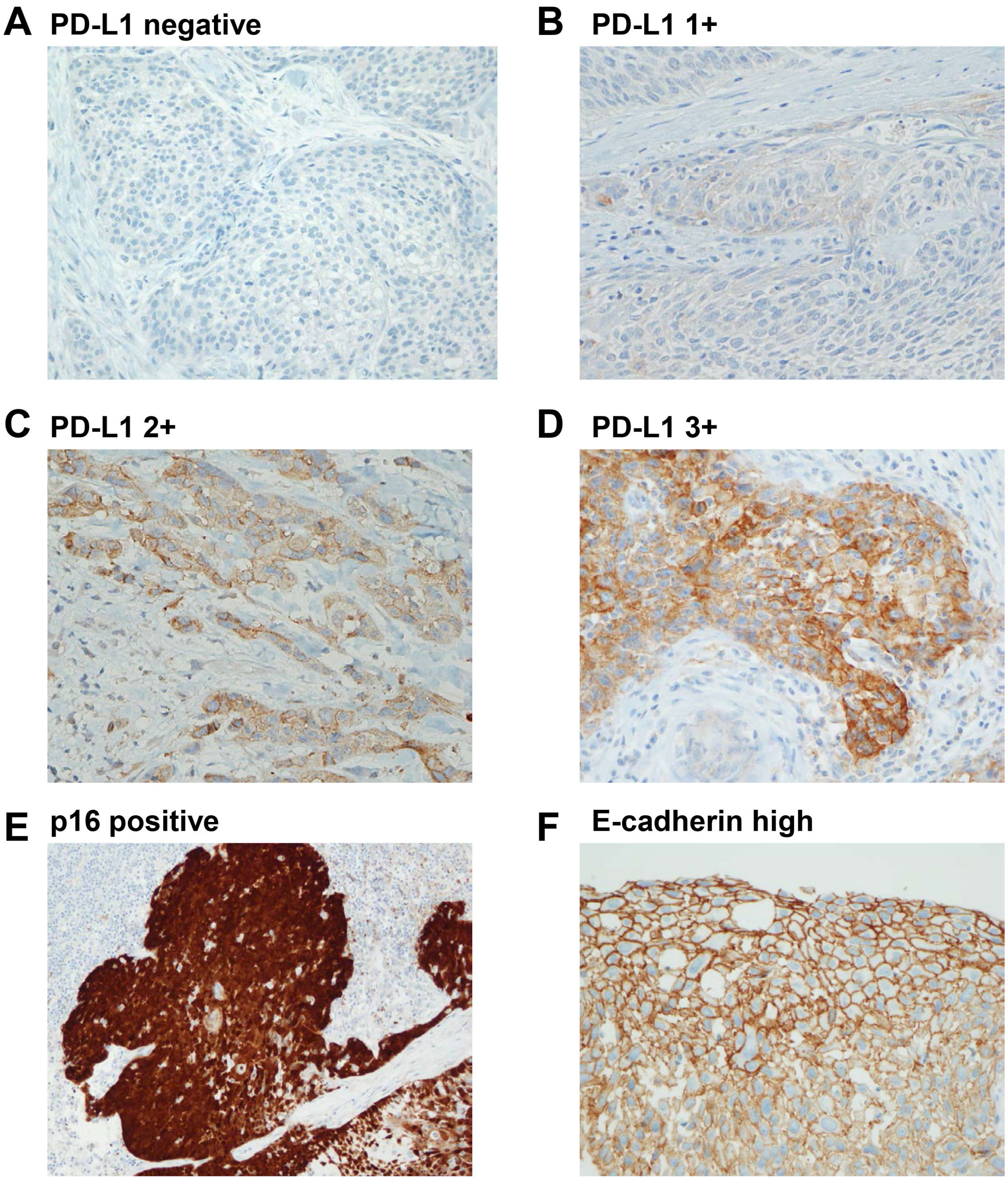

Supplementary Figure S1: Representative immunohistochemistry stain of PD-L1-negative. A. 1+ **B.** 2+ **C.** and 3+ **D.** (x400), as well as p16-positive **E.** (x200) and high E-cadherin expression **F.** (x400)

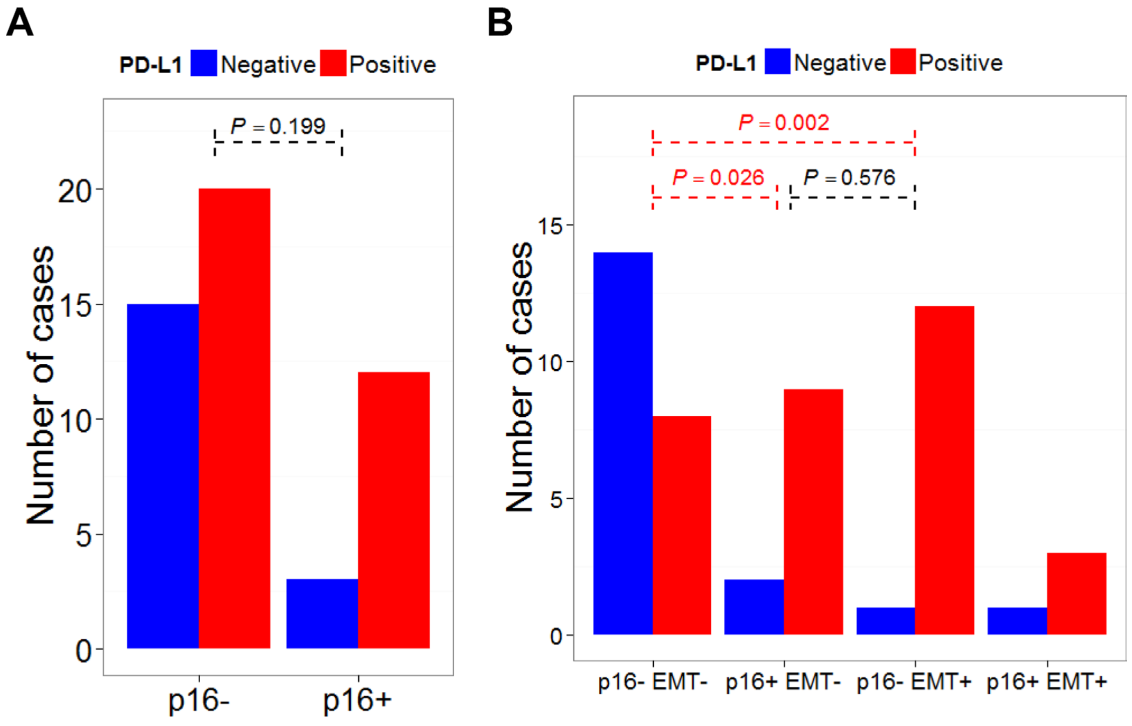

Supplementary Figure S2: PD-L1positivity according to p16 and epithelial-mesenchymaltransition (EMT) status. Number of cases with PD-L1 negative (blue bar) and positive (red bar) according to p16 **A.** and p16/EMTstatus **B.**

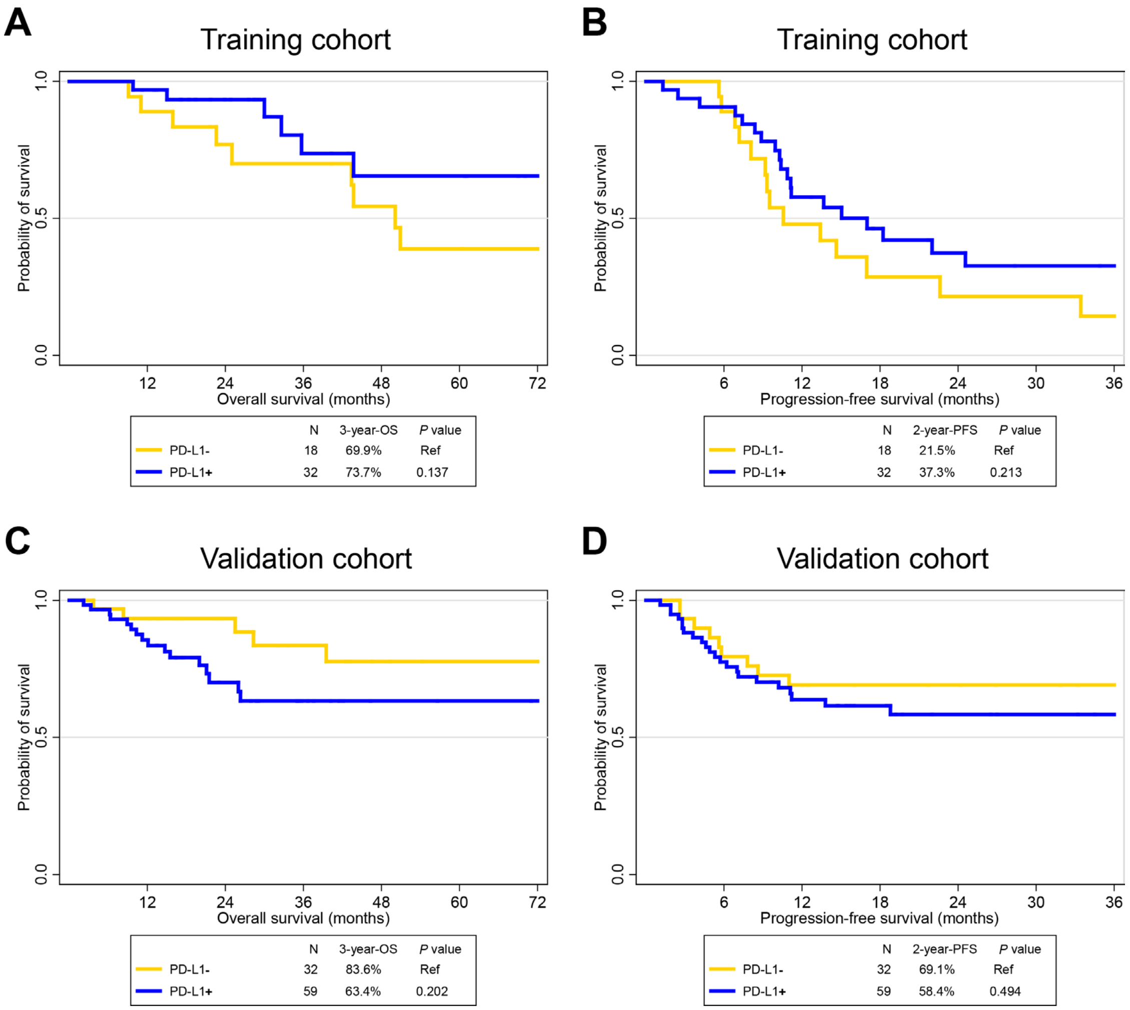

Supplementary Figure S3: Survival analysis according to PD-L1 in HNSCC patients of training (A, B) and validation cohorts (C, D). A Kaplan-Meier plot of overall survival (OS; A, C) and progression-free survival (PFS; B, D) according to PD-L1 statuses. Abbreviation: HNSCC, head and neck squamous cell carcinoma; Ref, reference.

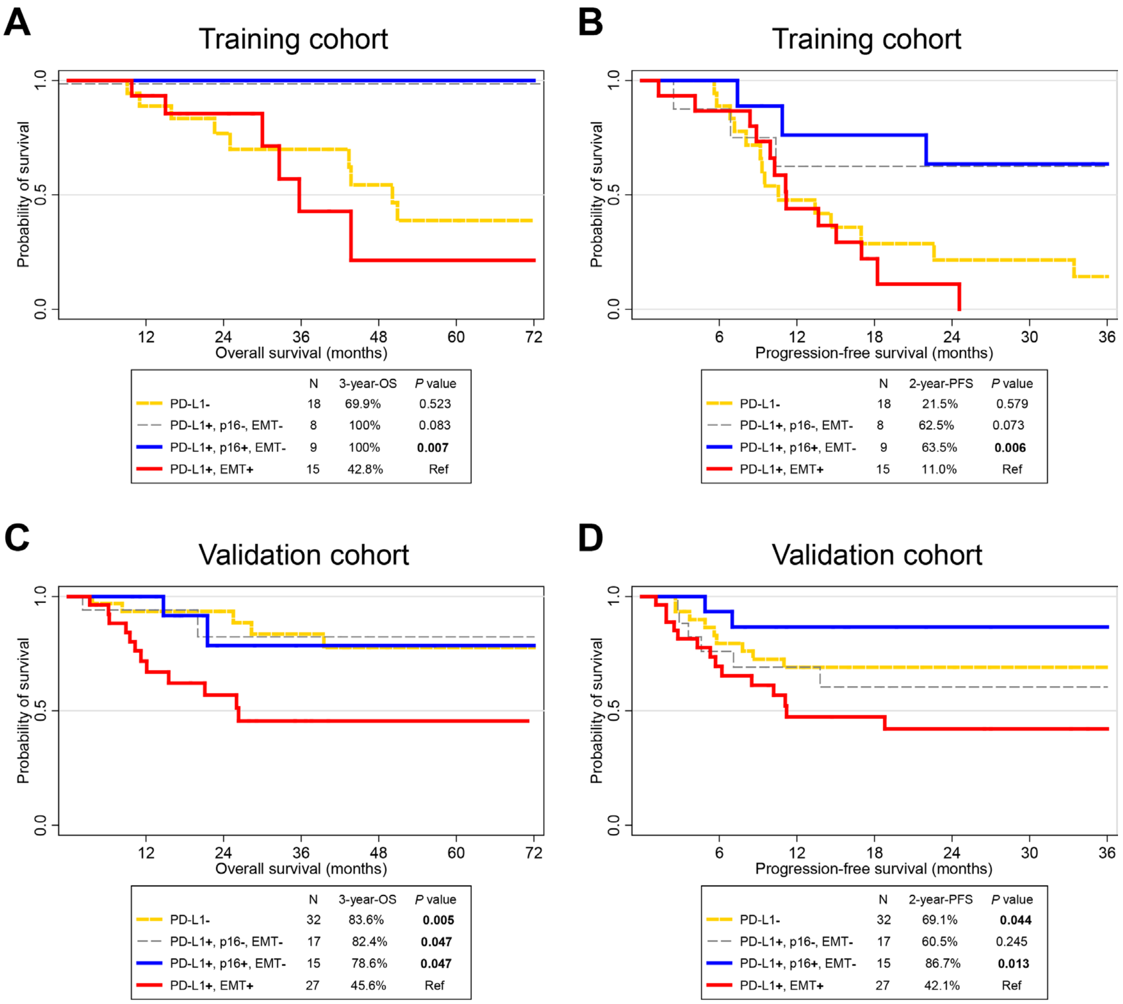

Supplementary Figure S4: Survival analysis according to PD-L1, p16, and epithelial-mesenchymal transition status in HNSCC patients of training (A, B) and validation cohorts (C, D). A Kaplan-Meier plot of overall survival (OS; A, C) and progression-free survival (PFS; B, D) according to PD-L1, p16, and epithelial-mesenchymal transition (EMT) status. Abbreviation: HNSCC, head and neck squamous cell carcinoma; Ref, reference.

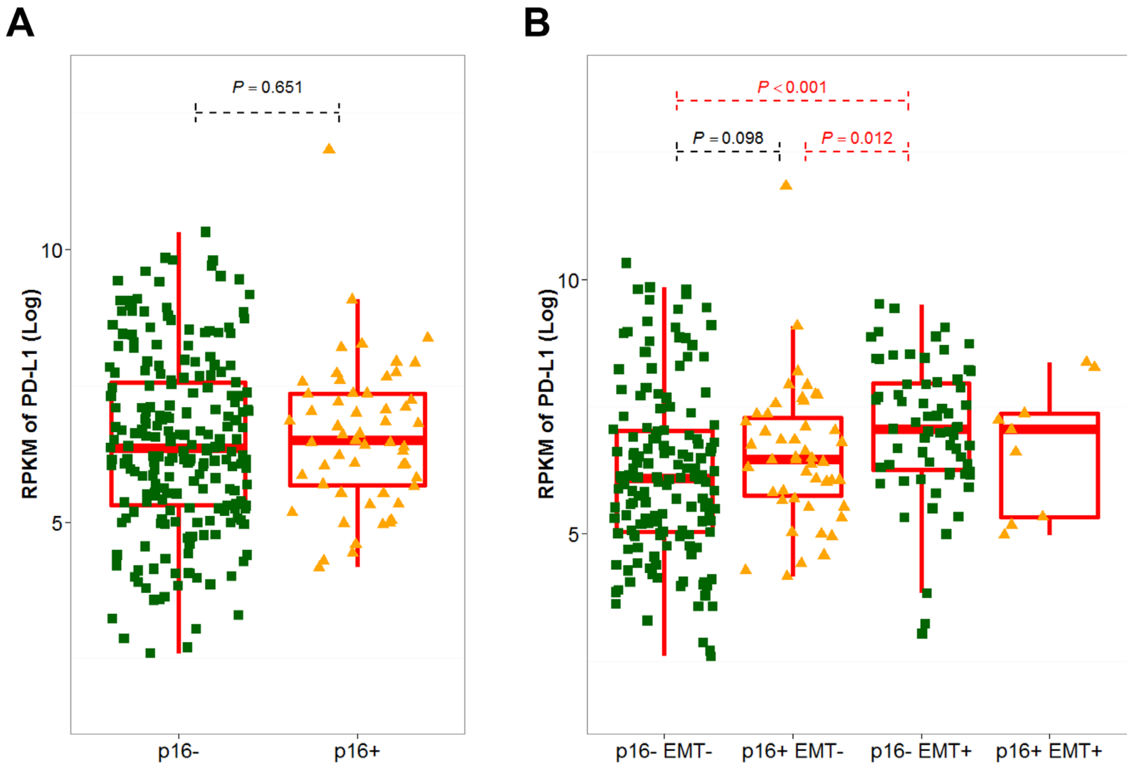

Supplementary Figure S5: PD-L1expression according to p16and EMTstatus in The Cancer Genome Altas (TCGA). PD-L1expression calculated by Log 2 value of its RPKMis compared according to p16status **A.** and p16/ EMTstatus **B.**

Supplementary Table S1: Epithelial-mesenchymal transition (EMT)-associated gene lists

See Supplementary File 1

Supplementary Table S2: Univariate analysis of factors affecting progression-free survival

		Training cohort				Validation cohort			
		Univariate		Multivariate		Univariate		Multivariate	
		HR (95% CI)	*P* value	HR (95% CI)	*P* value	HR (95% CI)	*P* value	HR (95% CI)	*P* value
Age	Continuous	1.02 (0.99-1.05)	0.092			1.00 (0.97-1.02)	0.830		
Sex	Female (vs. Male)	0.66 (0.28-1.60)	0.359			1.14 (0.57-2.27)	0.718		
Smoking	Yes (vs. No)	1.15 (0.58-2.27)	0.693			0.86 (0.40-1.84)	0.698		
ECOG	1 (vs. 0)	0.64 (0.30-1.33)	0.231			2.73 (1.41-5.29)	0.003	2.90 (1.49-5.62)	0.002
Stage	Continuous	1.19 (0.83-1.71)	0.336			0.94 (0.70-1.28)	0.707		
Location	Oropharynx (vs. Non-oropharynx)	0.53 (0.25-1.10)	0.089			0.60 (0.30-1.21)	0.153		
PD-L1	Positive (vs. Negative)	0.66 (0.34-1.28)	0.217			1.27 (0.64-2.54)	0.496		
p16	Positive (vs. Negative)	0.48 (0.22-1.04)	0.062			0.51 (0.24-1.09)	0.084		
EMT	Positive (vs. Negative)	1.99 (0.99-3.99)	0.051			2.06 (1.06-4.00)	0.033	2.24 (1.15-4.35)	0.018

Abbreviation: ECOG, Eastern Cooperative Oncology Group performance status; HR, hazard ratio; CI, confidence interval; EMT, epithelial-mesenchymal transition.
